# The Effects of FemmeBalance Supplement on Symptoms of Premenstrual Syndrome: A Four-Cycle Single-Arm Observational Study of a Novel Nutritional Supplement

**DOI:** 10.3390/life15091454

**Published:** 2025-09-17

**Authors:** Isabel Viña, Juan R. Viña

**Affiliations:** 1 IVB Wellness Lab, C/Colón 12, 46004 Valencia, Spain; 2 Departamento de Bioquímica y Biología Molecular, Facultad de Medicina, Instituto INCLIVA, Universitat de València, 46010 Valencia, Spain; juan.r.vina@uv.es

**Keywords:** premenstrual syndrome, FemmeBalance supplement, menstrual health, skin health, premenstrual symptoms screening tool

## Abstract

Background: Premenstrual syndrome (PMS) affects 950 million women worldwide. However, conventional pharmacological treatments offer limited improvements. Objective: This study aimed to evaluate the effectiveness of the FemmeBalance supplement in relieving PMS symptoms. Methods: We conducted a single-group study of 38 women, aged 18–40, with self-reported PMS symptoms. Participants received the FemmeBalance supplement (two capsules/day) for four menstrual cycles, completing the premenstrual symptoms screening tool (PSST) and study-specific questionnaires at baseline and on day 7 of each cycle. Skin changes were also assessed using dermatologist-graded facial photos. Results: By the first menstrual cycle, all PSST parameters showed significant improvement except insomnia. The greatest improvement was observed in interest in social activities, which improved by 40.13% in the fourth cycle. In addition, period heaviness was significantly decreased by the fourth cycle. Dermatologist skin grading showed improved overall skin health in 91.18% of participants. Moreover, 77.1% of the participants reported reduced PMS symptom severity, and 71.4% reported they would recommend the supplement. Conclusion: FemmeBalance was significantly effective in improving several PMS symptoms, including physical discomfort, mood disturbance, and skin health. The supplement also improved social functioning and overall menstrual health, suggesting that it could be an alternative to conventional PMS treatments.

## 1. Introduction

Premenstrual syndrome (PMS) affects nearly 950 million women worldwide, causing substantial health burdens, reduced quality of life, impaired workplace performance, and strained interpersonal relationships, with prevalence rising by 46.5% over the past three decades [1,2]. Approximately 55% of patients experience mood changes, and 92.7% report physical symptoms [3].

PMS is defined as a group of physical or psychological symptoms, such as lethargy and depressed mood, that occur during the last five days before menstruation, typically disappearing within four days after the onset of bleeding with no underlying organic cause [4]. However, PMS is considered a gynecological disorder according to the 11th edition of the International Statistical Classification of Diseases, while it is regarded as a psychiatric disorder according to the 5th edition of the Diagnostic and Statistical Manual of Mental Disorders (DSM-5) [5,6]. Premenstrual symptoms occur along a spectrum from mild forms with limited impact to severe cases that meet the criteria for premenstrual dysphoric disorder (PMDD), which causes marked functional impairment in work, social, and family life [7]. Polycystic ovary syndrome (PCOS) can mimic or exacerbate PMS symptoms, as both involve hormonal and neurochemical dysregulation. Women with PCOS have higher rates of PMS and PMDD, highlighting the importance of considering PCOS in differential diagnosis [8]. This discrepancy in classification highlights the ongoing debates over the pathogenesis and diagnosis of PMS, as evidenced by several theories proposed to explain its symptoms and the wide range of dietary, medical, and surgical approaches developed to manage these symptoms [9].

Evidence-based pharmacological treatments for PMS primarily include selective serotonin reuptake inhibitors (SSRIs) and specific, combined oral contraceptives. SSRIs have consistently demonstrated efficacy in reducing both mood and physical symptoms of PMS, with response rates of 60–75% in randomized controlled trials, with fluoxetine and sertraline being the most extensively studied [10,11]. Combined oral contraceptives containing drospirenone have shown superior efficacy compared to placebo in reducing premenstrual symptoms, particularly physical symptoms such as fluid retention and mood changes [12]. Standard pharmacological management, such as non-steroidal anti-inflammatory drugs (NSAIDs), is commonly used in PMS, but they focus only on decreasing pain [9]. Moreover, other managements, such as non-contraceptive estrogen-containing preparations, may fail to relieve PMS symptoms or even worsen them [13].

Nevertheless, these pharmacological treatments may present significant adverse effects, including gastrointestinal and sexual side effects of SSRIs and thromboembolic risk with oral contraceptives [9,14]. In this context, non-pharmacological therapies such as FemmeBalance represent a valuable alternative with a favorable safety profile, particularly for women seeking to avoid hormonal treatments or those experiencing intolerance to selective SSRIs.

In contrast, the ingredients in the FemmeBalance supplement, such as chasteberry fruit (*Vitex agnus-castus*) and curcumin, have shown promise in relieving PMS symptoms [15,16,17]. For instance, some studies have found that chasteberry fruit (*Vitex agnus-castus*) reduced pain by 50% in over half of the study participants, demonstrating its effectiveness in relieving PMS symptoms [8]. In addition, curcumin has shown significant efficacy in reducing PMS symptoms by inhibiting prostaglandin formation and modulating neurotransmitter levels [15,16]. Although not included in the traditional PMS diagnostic criteria, perimenstrual acne represents a well-documented hormonal manifestation affecting up to 65% of women, with 63% experiencing increased acne lesions during the late luteal phase compared to the follicular phase [18,19]. This condition occurs due to hormonal fluctuations, particularly androgen activity during the premenstrual period, leading to increased sebum production and inflammation [20]. Given that the FemmeBalance formulation contains N-acetylcysteine (NAC), which has demonstrated efficacy in acne treatment through its anti-inflammatory properties, including inhibition of inflammatory cytokines (TNF-α, IL-8, IL-6, IL-1β) and antimicrobial activity against acne-associated bacteria [21], dermatological assessment was included as a secondary exploratory outcome to evaluate potential skin benefits. In this context, this study aims to evaluate the effectiveness of the FemmeBalance supplement in relieving PMS symptoms, including pelvic pain, mood swings, acne, hair loss, breast tenderness, and others, using a participant questionnaire and skin expert assessment over four menstrual cycles.

Given the limited evidence for this specific multi-ingredient formulation and the established efficacy of individual components (chasteberry, curcumin, and NAC) in previous randomized trials, a single-arm observational design was selected as an appropriate initial approach to assess effectiveness signals and inform future randomized controlled trials.

## 2. Methods

### 2.1. Participants

Thirty-eight female participants aged 18–40 years were recruited for this study. All participants self-reported PMS symptoms. All participants met the specified inclusion and exclusion criteria. Sample size calculation for this single-group premenstrual syndrome study was performed using the R (pwr package, version 1.3-0). To estimate the sample size, a moderate effect size of approximately Cohen’s d of 0.5, a level of significance or type I error of 0.05 (5%), and a power or type II error or (1-β) of 0.8 (80%) were used, yielding a minimum required sample size of 34 patients.

### 2.2. Inclusion Criteria

The inclusion criteria were female participants aged 18–40 years with self-reported PMS symptoms for at least three consecutive months, including but not limited to pelvic pain, mood swings, acne, hair loss, fluid retention, breast tenderness, irritability, and episodes of crying or sadness. Eligible participants were generally healthy and not living with any uncontrolled chronic disease (excluding participants with chronic endocrine disorders such as PCOS), which could confound PMS symptom assessment due to overlapping hormonal and dermatological manifestations), regular menstrual cycles (21–35 days), and stable dietary habits, sleep schedules, and activity levels for at least 16 weeks, ensuring consistency with no significant dietary changes.

### 2.3. Exclusion Criteria

The exclusion criteria included chronic conditions limiting protocol adherence (e.g., oncological or psychiatric disorders), severe allergic reactions requiring EpiPen, and allergies to product ingredients. Women who were pregnant, breastfeeding, attempting conception, or unwilling or unable to comply with the protocol were excluded, as were those with premenstrual dysphoric disorder, endometriosis, reproductive malignancies, or peri-/postmenopausal status.

Additional exclusion criteria included the current use of hormonal birth control; any previous use within the last three months before the study; and the concurrent use of products, medications, or supplements specifically targeting PMS symptoms.

Participants were excluded from the study if they had undergone surgery or invasive procedures in the last six months, had experienced a major illness in the previous three months, or were currently undergoing or planning to undergo reproductive health and/or menstrual cycle-related procedures (such as hysterectomy, oophorectomy, or endometrial ablation) during the study period. Furthermore, participants experiencing amenorrhea or irregular menstrual cycles (longer than an average of 35 days), as well as those involved in or planning to join another trial during the study period, were also excluded. These menstrual cycle requirements effectively excluded participants with conditions such as PCOS, where irregular menstruation is a cardinal diagnostic feature affecting 70–80% of cases.

Finally, we excluded smokers, participants with a smoking history in the last three months, and those with a history of substance abuse.

### 2.4. Study Design

This was a virtual, single-group study. All participants were recruited during a single-day recruitment window: from 30 July to 31 July 2024. This brief recruitment window was designed to minimize temporal confounding variables and ensure all participants began the study under similar seasonal and environmental conditions, reducing potential baseline variations that could affect the interpretation of treatment effects on cyclical premenstrual symptoms. Patients with PMS were enrolled from Citruslabs (1639 11th Street, Santa Monica, CA, USA), specifically Citruslabs Research Team (Dr. Swathi Varanasi research group). They were informed about the study through explanation by a healthcare professional, ensuring that participation was voluntary and based on informed consent. Consequently, participation in the study was voluntary and anonymous.

In this study, participants were required to complete several questionnaires, including the premenstrual symptoms screening tool (PSST) [22] and study-specific questions, as well as submit photos of their facial skin. The PSST is a brief, retrospective questionnaire designed to identify women who experience clinical PMS or PMDD. The PSST translates the categorical diagnostic criteria from the DSM into a rating scale that assesses the severity and impact of premenstrual symptoms. It enables clinicians to quickly determine whether a woman meets the criteria for PMDD or severe PMS, facilitating timely intervention. The tool is recognized for its practicality, reliability, and ease of use, making it suitable for both clinical and research settings [22].

Before enrollment, participants received consent forms detailing the study process, instructions, evaluation methods, and bill of rights. After completing the consent process, participants completed the baseline questionnaire and took baseline photos of their skin. Following this, they began the FemmeBalance supplement, with a dosage of two capsules daily during their evening meal.

Subsequent questionnaires were completed on day 7 of the participants’ first, second, third, and fourth menstrual cycles. On day 7 of their fourth cycle, participants also submitted photos of their facial skin. The final questionnaire and photo on day 7 of their fourth cycle marked the end of the study.

### 2.5. Intervention

The FemmeBalance supplement (IVB Wellness Lab) contains the following active ingredients: 342 mg of dry extract of Sabal serrulata fruit (*Serenoa repens*), 103 mg of fatty acids, 300 mg of N-acetylcysteine (NAC), 250 mg of dry extract of turmeric rhizome (*Curcuma longa*), 237 mg of curcuminoids, 183 mg of curcumin, 240 mg of dry extract of chasteberry fruit (*Vitex agnus-castus* L., VAC), 1.2 mg of agnuside, 4.94 mg of pantothenic acid (as D-Pantothenate calcium), 5 mg of dry extract of black pepper fruit (*Piper nigrum*), and 4.75 mg of piperine.

Patients took the intervention daily with the FemmeBalance supplement, from day 1 of the study until day 7 of the fourth menstrual cycle.

### 2.6. Dermatologist Grading

Dermatological assessment was included as a secondary exploratory outcome based on the established association between perimenstrual acne exacerbation and hormonal fluctuations, combined with the documented anti-acne properties of N-acetylcysteine (NAC) present in the FemmeBalance formulation. This evaluation aimed to assess potential skin benefits beyond traditional PMS symptoms.

Participants submitted photographs of their faces at baseline and day 7 of their fourth menstrual cycle for virtual skin grading. A dermatologist analyzed these images to assess any improvement from the baseline. The evaluated parameters included overall skin health, fine lines/wrinkles, dryness, background redness, brightness, inflammation, and hormonal acne.

### 2.7. Data Analysis

Data from the PSST were collected using a 4-point Likert scale for each question. Responses were converted into numerical values ranging from 1 to 4, with 4 indicating the worst outcome and 1 indicating the best outcome. The PSST consists of two main questions, each with several sub-questions that were analyzed independently.

Data from the study-specific questions were collected using a 5-point Likert scale, with textual data converted into numerical values ranging from 1 to 5. For the 10 questions regarding the negative effects of PMS, a score of 1 indicated the best outcome, while a score of 5 indicated the worst outcome. For the five questions regarding positive health parameters, a score of 1 represented the worst outcome, whereas 5 represented the best outcome.

Statistical analyses were conducted on all available data using the intention-to-treat (ITT) approach. Data were assessed for normality using the Pearson test or the Shapiro–Wilk test when the sample size was insufficient for the Pearson test. A mixed-effects analysis with Dunnett’s multiple comparison test was used for statistical analysis. Statistical analysis was conducted using GraphPad Prism 10.4, with the significance level set at a *p*-value of 0.05.

Expert skin grading was presented as the percentage of participants classified by the dermatologist as having improved from the baseline. The data were not statistically analyzed. For the study-specific questions, evaluated only on day 7 of the first, second, third, and fourth cycles, results were presented as the percentage of participants who agreed with each question.

## 3. Results

### 3.1. Characteristics of Patients

We successfully recruited 39 patients, resulting in an 86% statistical power, which exceeds the conventional 80% threshold and is well-justified based on similar PMS intervention studies [23]. The mean age was 32.2 ± 4.8 years. Demographic characteristics of patients are shown in Table 1. All participants reported adherence to the supplementation protocol, although compliance was based on self-reporting rather than objective measures. None of the participants reported discontinuing supplements during the study period.

### 3.2. Impact of the Test FemmeBalance Supplement on Premenstrual Symptoms Evaluated by the PSST Questionnaire

**Figure 1:**
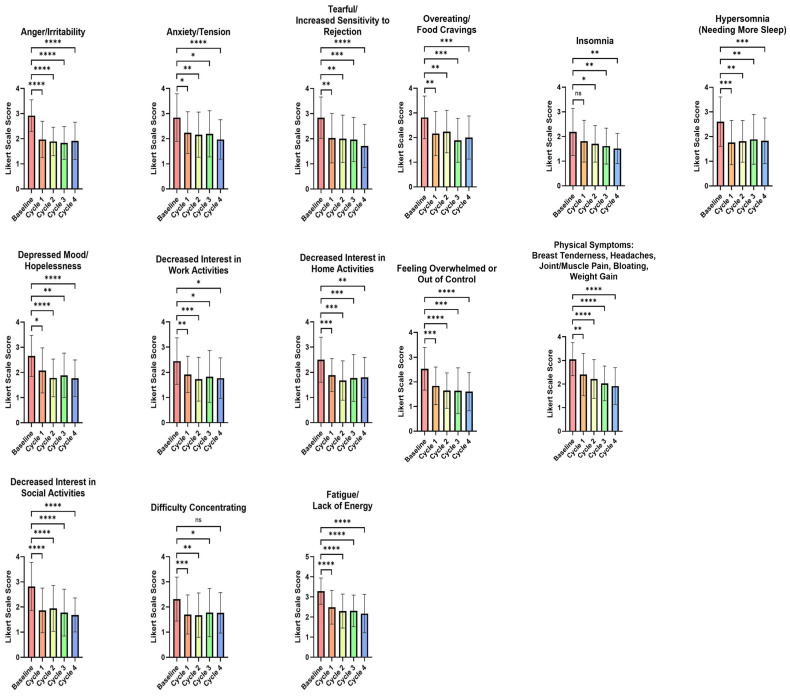
Change in premenstrual symptoms evaluated by the PSST questionnaire from baseline across 4 menstrual cycles. Data is graphed as group means and standard deviations. A decrease in score indicates an improvement. ns = *p* > 0.05, * = *p* < 0.05, ** = *p* < 0.01, *** = *p* < 0.001, **** = *p* < 0.0001.

The effect of the FemmeBalance supplement on 14 premenstrual symptoms was assessed using the PSST questionnaire (see Figure 1). All parameters showed significant differences from baseline, except for insomnia during the first menstrual cycle. These included anger/irritability (*p* < 0.0001), depressed mood/hopelessness (*p* = 0.0138), and physical symptoms (e.g., breast tenderness and bloating) (*p* = 0.0025). The greatest improvement was seen in the parameter “Decreased interest in social activities,” which showed a reduction of 33.77% from the baseline (2.8 vs. 1.8, *p* < 0.0001).

Additionally, all 14 parameters showed significant differences from the baseline in the second and third menstrual cycles. By the fourth cycle, 13 of the 14 parameters were significantly different from the baseline, including anxiety/tension (*p* < 0.0001), fatigue/lack of energy (*p* < 0.0001), and overeating/food cravings (*p* = 0.0007). The most significant improvement was again observed in the “Decreased interest in social activities” parameter, showing a 40.13% reduction from the baseline (Supplementary Table S1, Figure 1).

**Figure 2:**
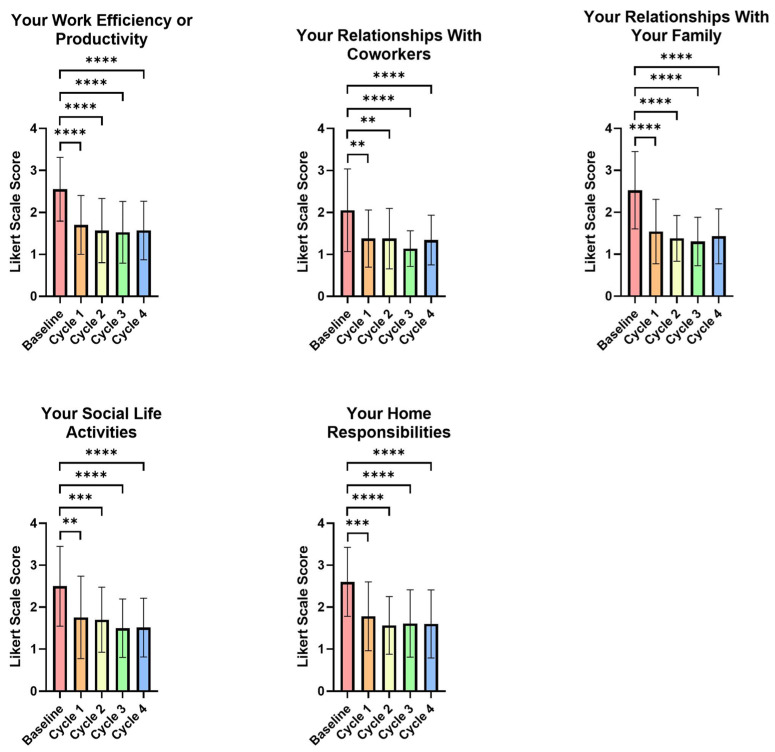
Visual representation of premenstrual symptom interference with daily activities and relationships evaluated by the PSST questionnaire. Data is graphed as group means and standard deviations. A decrease in score indicates an improvement. ** = *p* < 0.01, *** = *p* < 0.001, **** = *p* < 0.0001.

The effects of PMS symptoms on the five parameters were assessed using the PSST questionnaire. By the first menstrual cycle, all five parameters showed significant improvements compared to the baseline, including reduced PMS symptoms that interfered with work productivity (*p* < 0.0001), co-worker relationships (*p* = 0.0026), family relationships (*p* < 0.0001), social life activity (*p* = 0.0016), and home responsibilities (*p* = 0.0001) (see Supplementary Table S2, Figure 2). These improvements remained significant for the second, third, and fourth menstrual cycles (see Supplementary Table S2, Figure 2).

### 3.3. Impact of the FemmeBalance Supplement on PMS Symptoms Evaluated by Study-Specific Questionnaires

**Figure 3:**
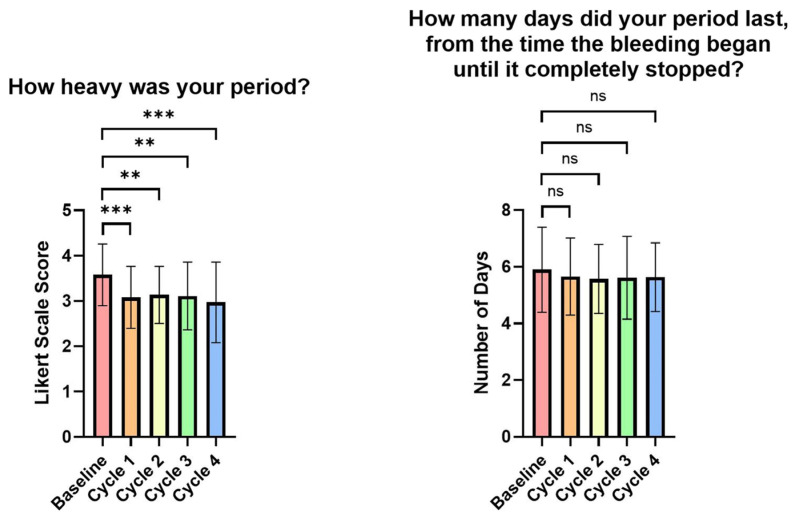
Visual representation of heaviness and length of period evaluated by study-specific questionnaires. Data is graphed as group means and standard deviations. A decrease in score indicates an improvement. ns = *p* > 0.05, ** = *p* < 0.01, *** = *p* < 0.001.

Participants were asked to answer questions to assess the heaviness and length of their period compared to the last period. By the first menstrual cycle, there was a significant decrease in reported period heaviness (*p* = 0.0005). This decrease remained significant for the second (*p* = 0.0019), third (*p* = 0.007), and fourth menstrual cycles (*p* = 0.0004). However, no significant differences in the period length were observed across these time points (see Supplementary Table S3, Figure 3).

Menstrual cycle length remained within the 21–35 days range across participants, and no new menstrual irregularities were reported during follow-up. However, only the self-reported heaviness and length were recorded.

Participants were asked 10 questions about negative PMS symptoms and 5 questions about their overall health compared to the week before and during their last menstrual period. By the first menstrual cycle, all PMS parameters significantly improved compared to baseline, except for the severity of hormonal hair loss.

**Figure 4:**
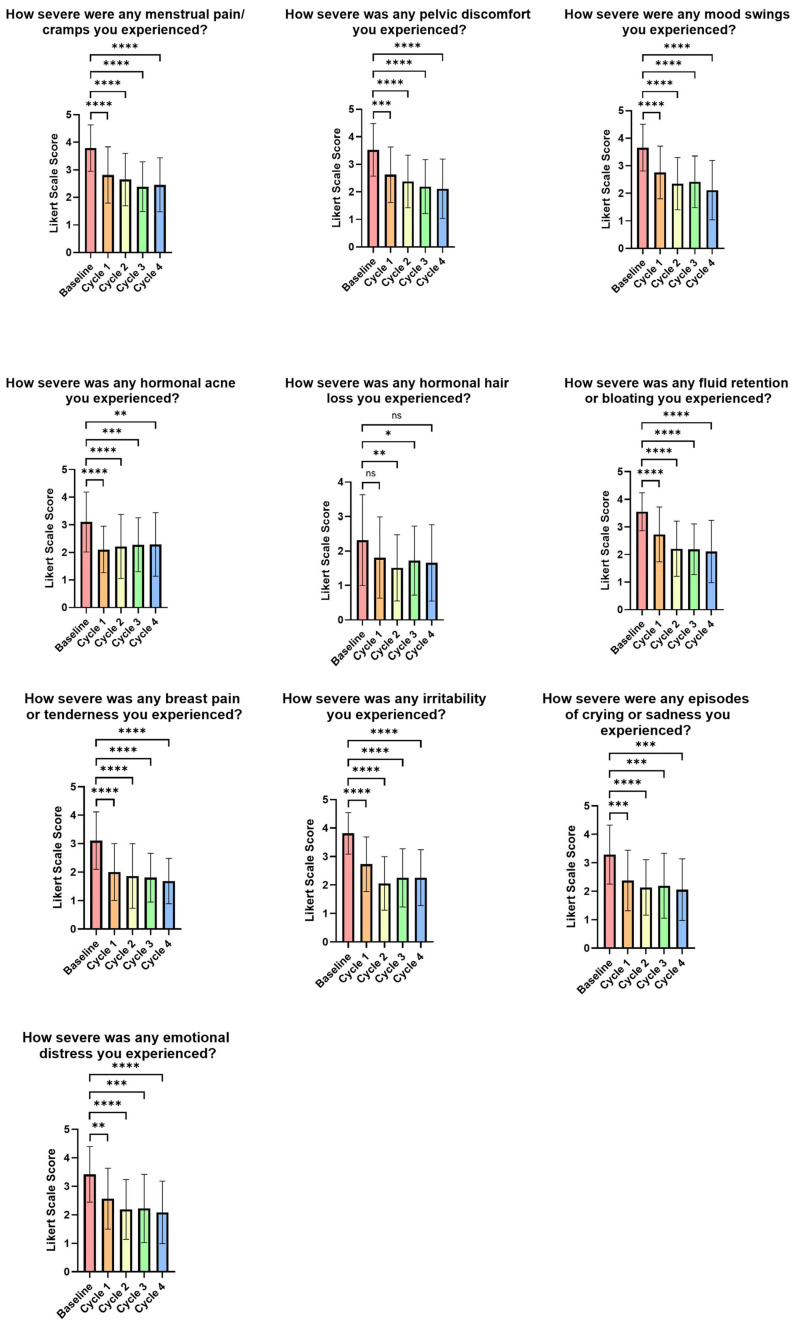
Visual representation of premenstrual symptoms evaluated by study-specific questionnaires. Data is graphed as group means, and standard deviations are shown. A decrease in score indicates an improvement. ns = *p* > 0.05, * = *p* < 0.05, ** = *p* < 0.01, *** = *p* < 0.001, **** = *p* < 0.0001.

These PMS parameters included the severity of menstrual pain/cramps (*p* < 0.0001), mood swings (*p* < 0.0001), and breast pain or tenderness (*p* < 0.0001). By the second menstrual cycle, all 10 parameters showed significant improvements compared to baseline, including the severity of hair loss (*p* = 0.0019), hormonal acne (*p* < 0.0001), and irritability (*p* < 0.0001). Additionally, the third menstrual cycle showed significant improvement across all parameters compared to the baseline, including the severity of pelvic discomfort (*p* < 0.0001), episodes of crying or sadness (*p* = 0.0002), and emotional distress (*p* = 0.0002). However, the fourth menstrual cycle showed similar results, except for the severity of hormonal hair loss. The greatest improvement was observed in breast pain or tenderness, which showed a 45.71% reduction from baseline (see Supplementary Table S4, Figure 4).

**Figure 5:**
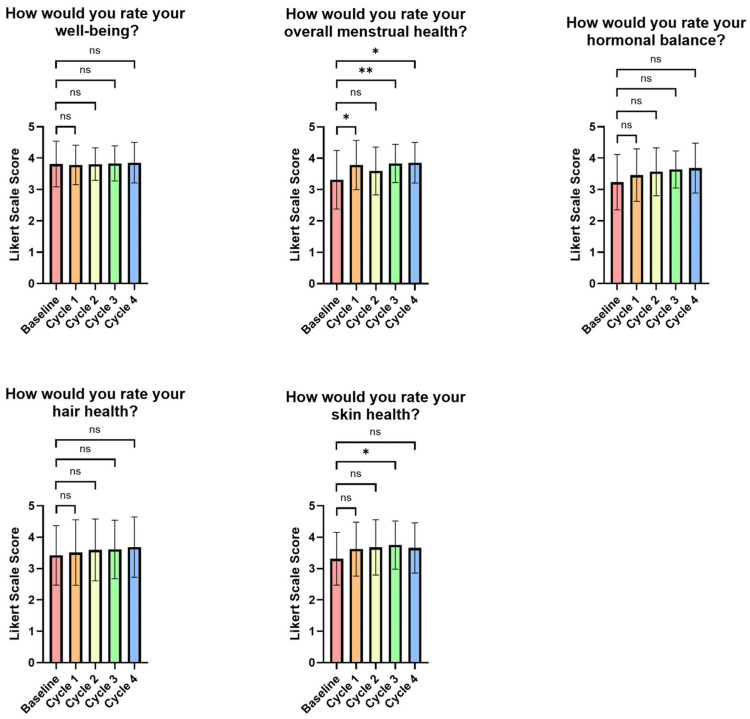
Visual representation of premenstrual symptoms evaluated by study-specific questionnaires. Data is graphed as group means, and standard deviations are shown. An increase in scores indicates an improvement. ns = *p* > 0.05, * = *p* < 0.05, ** = *p* < 0.01.

However, by the first menstrual cycle, only overall menstrual health showed significant improvement among the five overall health parameters (*p* = 0.017) (see Figure 5). Moreover, none of the overall health parameters showed any significant differences by the end of the second menstrual cycle. The third menstrual cycle showed significant improvements in both overall menstrual and skin health compared to baseline (*p* = 0.005 and *p* = 0.0212, respectively), whereas the fourth menstrual cycle showed significant improvement only in the overall menstrual health parameter (*p* = 0.0353) (see Supplementary Table S5, Figure 5).

### 3.4. Impact of the FemmeBalance Supplement on Expert Skin Grading

By day 7 of the fourth menstrual cycle, 91.18% of participants demonstrated an improvement from baseline in overall skin health, 32.35% in fine lines and wrinkles, 70.59% in dryness, 64.71% in background redness, 82.35% in brightness, and 41.18% in inflammation and hormonal acne (see Table 2).

### 3.5. Participants’ Perception of the FemmeBalance Supplement and Its Impact on Relieving PMS Symptoms

Participants were asked to respond to questions on day 7 of the first, second, third, and fourth cycles to evaluate their perceptions of the supplemental and its effects on PMS symptoms (see Table 3). Participants answered these supplement evaluation questions using a scale ranging from “strongly disagree” to “strongly agree. “ The responses “strongly agree” and “agree” were merged into a single “combined agree” outcome to assess overall agreement better. Combined agreement outcomes that showed ≥65% were considered “notable” positive responses.

No notable positive responses were observed for any of the questions at the first menstrual cycle (see Table 3). Only three of the 15 parameters showed a notable positive response by the second menstrual cycle, including reduced severity of PMS symptoms (67.6%), irritability (67.6%), and improved well-being (73.0%) (see Table 3). Additionally, only four of the 15 parameters showed a notable positive response by the third menstrual cycle, with 75% of the participants reporting reduced severity of menstrual pain or cramps and 80.6% reporting less severe pelvic discomfort since using the supplement (80.6%) (Table 3). However, nine of the 15 parameters showed a notable positive response by the fourth menstrual cycle, including reduced severity of PMS symptoms (77.1%) and improved overall menstrual health (74.3%). Finally, 71.4% of participants agreed they would recommend the supplement to their friends and family, and 71.4% would like to continue using it.

## 4. Discussion

PMS is a multifactorial syndrome that often presents with physical and psychological symptoms, necessitating the use of supplements that combine several ingredients to address these various aspects of PMS. Our study showed that the FemmeBalance supplement improved physical PMS symptoms, including breast tenderness, headaches, joint/muscle pain, bloating, and weight gain, through four menstrual cycles (<0.0001). Additionally, this supplement improved psychological PMS symptoms, including anxiety/tension (*p* < 0.0001), fatigue/lack of energy (*p* < 0.0001), and overeating/food cravings (*p* = 0.0007).

It is important to note that most participants in our study reported mild to moderate PMS symptoms at the baseline. Although statistical improvements were observed across nearly all domains, the clinical significance of these changes should be interpreted cautiously. Small numerical changes in symptom scores, although statistically significant in a relatively homogeneous cohort, may not equate to meaningful relief in women with more severe PMS or PMDD. This raises the possibility that the observed improvements reflect enhanced tolerability and functional well-being in mild cases, whereas the benefit–risk profile in more severe cases remains untested.

FemmeBalance should be considered within the category of non-pharmacological PMS treatments. Its main components (*Vitex agnus-castus* (VAC), curcumin, and N-acetylcysteine) have shown benefits in randomized trials. Recent meta-analyses confirm that Vitex is superior to placebo but not consistently superior to other comparators, with modest effect sizes, and VAC preparations were confirmed to be effective in the reduction of PMS symptoms [24,25]. In a study by Schellenberg et al., a 50% reduction in PMS symptoms occurred in over half of the participants, with significant improvement in those receiving 20 mg of VAC compared to placebo (*p* < 0.0001) [17]. Similarly, Ma et al. conducted a double-blind trial of 67 patients using the PMSD tool [26]. VAC improved 16 of the 17 symptom domains, except abdominal cramps, consistent with PMS characteristics [26]. Curcumin has also been shown to significantly reduce PMS severity [27]. N-acetylcysteine (NAC) has been shown to be beneficial for PMS and PMDD through antioxidant and anti-inflammatory mechanisms [28]. Our findings suggest that the multi-ingredient formulation in FemmeBalance provides superior clinical outcomes compared to single-component approaches through synergistic mechanisms. While meta-analyses have shown that Vitex alone has modest effect sizes, our combination leverages complementary pathways: Vitex targets dopaminergic and hormonal regulation, curcumin addresses inflammatory cascades, and N-acetylcysteine provides antioxidant and anti-inflammatory mechanisms. This multi-target approach may explain why we achieved significant improvements across 91.18% of participants in skin health and substantial functional improvements in social activities (40.13% improvement by cycle 4).

Moreover, our study also found improvement in PMS-related headaches by the fourth cycle, aligning with Ambrosini et al., who treated 100 women with PMS and migraine with 40 mg VAC for three months [29]. They reported marked improvement in 66% of patients, mild improvement in 26%, and no change in 8% [15]. Moreover, 42% showed a 50% reduction in monthly migraine attacks [29]. These findings may be related to the dopaminergic action of VAC and its affinity for central opioid receptors, particularly subtypes m and k [30].

Understanding the association between both serotonin and brain-derived neurotrophic factor (BDNF) and PMS symptoms is crucial in explaining the effect of curcumin on PMS symptoms [15]. Serotonin influences the psychological symptoms of PMS, especially the development of behavioral symptoms (irritability and aggression), mood symptoms (anxiety and depression), and a reduced pain threshold [11,31,32]. Cubeddu et al. reported lower BDNF levels in the luteal phase of women with PMS, suggesting a role in symptom development [33]. Similarly, Fanaei et al. found that in 70 women randomized to curcumin or placebo, symptom reduction correlated with higher serum BDNF [15].

Our supplement contained 237 mg of curcuminoids, 183 mg of curcumin, and 4.75 mg of piperine. This combination was also used by Karbasi et al., who randomized 80 participants with dysmenorrhea and PMS to receive either a placebo or 500 mg of curcuminoids and 5 mg of piperine, 7 days before menstruation and for three menstrual cycles [34]. They found that curcumin significantly increased the ability to neutralize free radicals compared to placebo (*p* = 0.031) [34].

These results explain the improved PMS symptoms in our study, as inflammation and oxidative stress are associated with both physical and psychological symptoms of PMS, and there is a strong correlation between these symptoms and interleukin levels, particularly interleukin and interleukin-6 [35].

NAC, another ingredient of our supplement, also acts as an antioxidant because acetylated cysteine can cross the blood–brain barrier and elevate glutathione levels, which is the main antioxidant inside the brain [36]. Thus, NAC directly or indirectly influences the inflammatory process by neutralizing the oxidative stress caused by free radicals [37]. Rajabi et al. randomized 119 women to 450 mg of NAC, 10 mg of fluoxetine, or placebo for two menstrual cycles to assess PMS symptoms using the daily record of severity of problems (DRSP) and Hamilton scores. They found that NAC and fluoxetine significantly improved PMS symptoms compared to placebo (*p* < 0.001) [29].

Our study found self-reported hair loss severity improved significantly during the second and third cycles, with reductions of 34.64% and 25.63% compared to baseline. However, no significant improvements were observed during the first and fourth cycles. These inconsistent outcomes suggest that the potential effect of FemmeBalance on hair health remains inconclusive and warrants further investigation in longer trials with larger cohorts. These results may be due to *Serenoa repens*, another ingredient in our supplement that was found to increase total hair count and resistance to traction after 16 weeks of taking 50 mg of *Serenoa repens* [38]. On a cellular level, the lipostatic extract of *Serenoa repens* improved keratinocyte proliferation, hair regeneration, and follicle growth while reducing hair loss via mitochondrial pathways and the activation of tumor necrosis factors [39,40].

Our study found that 91.18% of the participants improved their overall skin health. Improvements were also observed in terms of dryness (70.59%), brightness (82.35%), and hormonal acne (41.18%). These results could be attributed to NAC, which reduced the number of acne lesions in a controlled, double-blind study of 65 patients who used 5% NAC gel for 8 weeks [41]. In addition to its ability to neutralize free radicals, NAC can damage microbial biofilms involved in acne pathology, such as Propionibacterium, which release lysosomal enzymes that not only disrupt the follicular epithelium but also trigger further inflammation [42,43,44]. NAC exerts its antimicrobial action through (1) competitive inhibition of cysteine consumption, (2) sulfhydryl-mediated interaction with bacterial proteins, and (3) neutralization of oxidative stress that results from lysosomal enzymes [45].

### Strengths and Limitations

This study used a validated tool, such as the PSST [23], to assess PMS symptoms, which improved the reliability of our study and allowed cross-study comparability. Additionally, we used dermatology expert skin grading, which added objective measures of skin-related outcomes. We also used an ITT analysis method that reduced attrition bias. However, this was a non-randomized, single-group study, which may limit the causal interpretation of our results. The absence of a control group, especially a placebo group, makes it difficult to dismiss other impacts, such as the placebo effect, natural symptom changes over time, or even observation influence effects. To more reliably confirm the current findings and establish a direct causal relationship between FemmeBalance supplementation and the observed improvements in premenstrual syndrome (PMS) symptoms and skin health, future research should ideally adopt a randomized controlled trial (RCT) design, preferably including a placebo-controlled arm. The absence of statistical analysis for expert-assessed skin ratings represents a key limitation, as it prevents drawing definitive conclusions about the significance of the observed improvements in this objective measure. This methodological decision was based on treating dermatological assessments as secondary, descriptive outcomes rather than formal hypothesis-testing endpoints, given the exploratory nature of this pilot study and the prioritization of statistical power for primary PMS symptom outcomes measured by validated tools. To strengthen the interpretability and credibility of such findings, future studies should incorporate appropriate statistical evaluation of these clinician-rated outcomes. Another limitation is the absence of formal hormonal testing and comprehensive gynecological evaluations, which represents a limitation of our screening protocol, as subclinical hyperandrogenic conditions or PCOS with currently regular cycles might have gone undetected despite our multi-layered exclusion criteria. Additionally, the nature of the responses, outcomes measurement, and self-reported PMS symptoms can introduce recall bias. Although perimenopausal women were excluded based on age and menstrual history, hormonal assays were not performed to confirm menopausal status. This limitation could have allowed for misclassification in a small subset of participants, although the probability is low given the age distribution. Furthermore, the study relied on self-reported compliance with supplement intake, which may introduce adherence bias and potentially affect the accuracy of dose–response relationships.

## 5. Conclusions

FemmeBalance supplementation significantly improved PMS symptoms, including physical and psychological symptoms, and improved overall skin health. The supplement also improved social functioning and overall menstrual health, suggesting it may be an alternative to conventional PMS treatments.

## Figures and Tables

**Table 1:** Baseline demographic and clinical characteristics of study participants (N = 39).

Characteristics	Patient Values
Age (mean ± SD)	32.2 ± 4.8
Weight in pounds (mean ± SD)	165.9 ± 39.8
Weight in Kg (mean ± SD)	75.2 ± 18.1
Fitzpatrick Scale Rating	
Type 1 (%)	15.4%
Type 2 (%)	41.0%
Type 3 (%)	35.9%
Type 4 (%)	5.1%
Type 5 (%)	2.6%

Data presented as mean ± standard deviation for continuous variables and percentages for categorical variables. Fitzpatrick Scale Rating indicates skin phototype classification (Type 1: very fair skin that always burns; Type 2: fair skin that usually burns; Type 3: medium skin that sometimes burns; Type 4: olive skin that rarely burns; Type 5: dark skin that very rarely burns). All participants met inclusion criteria of regular menstrual cycles (21–35 days) and were generally healthy with no chronic endocrine disorders.

**Table 2:** Descriptive analysis of changes in facial skin health and appearance parameters as determined by expert skin grading.

Parameter	% of Group Dermatologist Rated as Improved from Baseline (*n*)
Overall Skin Health	91.18% (31)
Fine Lines/Wrinkles	32.35% (11)
Dryness	70.59% (24)
Background Redness	64.71% (22)
Brightness	82.35% (28)
Inflammation and Hormonal Acne	41.18% (14)

The percentage of participants that the dermatologist classified as having improved from baseline at each time point. Total number of patients (*n*) = 34. Data are presented as descriptive percentages of participants rated as improved by the dermatologist. Statistical analysis was not performed for these expert-rated outcomes, as they were designed as secondary, descriptive endpoints to provide a supplementary clinical context. Dermatological assessments were based on qualitative clinical judgments, which are more suitable for descriptive rather than inferential statistical analysis.

**Table 3:** Longitudinal assessment of participant-reported outcomes and product satisfaction across four menstrual cycles.

How Much Do You Agree with the Following:	Cycle 1 (*n* = 37)	Cycle 2 (*n* = 37)	Cycle 3 (*n* = 36)	Cycle 4 (*n* = 35)
My PMS symptoms are less severe since using this product.	54.1%	67.6%	77.8%	77.1%
My hormonal balance has improved since using this product.	40.5%	59.5%	58.3%	60.0%
My overall menstrual health has improved since using this product.	45.9%	64.9%	63.9%	74.3%
My well-being before and during my period has been better since using this product.	56.8%	73.0%	72.2%	68.6%
My hair health has improved since using this product.	24.3%	37.8%	27.8%	48.6%
My skin health has improved since using this product.	32.4%	48.6%	50.0%	54.3%
I have experienced less severe menstrual pain or cramps since using this product.	59.5%	64.9%	75.0%	68.6%
I have experienced less severe pelvic discomfort since using this product.	51.4%	64.9%	80.6%	71.4%
I have experienced less severe hormonal mood swings since using this product.	45.9%	64.9%	63.9%	65.7%
I have experienced less hormonal hair loss since using this product.	24.3%	37.8%	36.1%	42.9%
My hormonal bloating or fluid retention has reduced or resolved since using this product.	35.1%	43.2%	55.6%	60.0%
I have experienced less severe hormonal breast pain or tenderness since using this product.	48.6%	54.1%	63.9%	68.6%
I feel less irritable before and during my period since using this product.	51.4%	67.6%	61.1%	62.9%
I have experienced fewer episodes of crying or sadness before and during my period since using this product.	56.8%	59.5%	61.1%	71.4%
I am less prone to emotional distress before and during my period since using this product.	45.9%	62.2%	63.9%	68.6%
I would like to continue using this product.	-	-	-	71.4%
I would purchase this product.	-	-	-	62.9%
I would recommend this product to family and friends.	-	-	-	71.4%

Data represent the combined percentage of participants who responded “strongly agree” or “agree” to each statement (Combined Agree outcomes). Values ≥ 65% are highlighted in blue and considered “notable” positive responses, indicating clinically meaningful participant satisfaction. Sample sizes: Cycle 1 (*n* = 37), Cycle 2 (*n* = 37), Cycle 3 (*n* = 36), Cycle 4 (*n* = 35). Questions regarding product continuation, purchase, and recommendation were only assessed in Cycle 4. Assessment was conducted on Day 7 of each menstrual cycle using a Likert scale ranging from “strongly disagree” to “strongly agree”. Missing responses due to participant dropout or missed assessments are excluded from percentage calculations.

## Data Availability

All data relevant to the study are included in the article or uploaded as online Supplemental Information. Data may be available upon reasonable request from the corresponding author.

## Supplementary Materials

The following supporting information can be downloaded at https://www.mdpi.com/article/10.3390/life15091454/s1, Table S1: Effect of the FemmeBalance Supplement on Premenstrual Symptoms Evaluated by the PSST Questionnaire; Table S2: Effect of the FemmeBalance Supplement on Premenstrual Symptom Interference with Daily Activities and Relationships Evaluated by the PSST Questionnaire; Table S3: Effect of the FemmeBalance Supplement on Heaviness and Length of Period Evaluated by Study Specific Questionnaires; Table S4: Effect of the FemmeBalance Supplement on Premenstrual Symptoms Evaluated by Study-Specific Questionnaires; Table S5: Effect of the FemmeBalance Supplement on Premenstrual Symptoms Evaluated by Study-Specific Questionnaires.
