# Supplementary material for: The Effects of FemmeBalance Supplement on Symptoms of Premenstrual Syndrome: A Four-Cycle Single-Arm Observational Study of a Novel Nutritional Supplement
Source: Life (Basel). 2025 Sep 17;15(9):1454. doi: 10.3390/life15091454 (PMC12471523; doi:10.3390/life15091454)
Supplement: Supplementary file 1 [file life-15-01454-s001.zip › life-3835846-supplementary.pdf]

Table S1: Effect of the FemmeBalance Supplement on Premenstrual Symptoms Evaluated by the PSST Questionnaire.

During your most recent period, did you experience any of the following premenstrual symptoms, which started before your period and stopped within a few days of bleeding?	Baseline (n=38)		Cycle 1 (n=37)				Cycle 2 (n=37)				Cycle 3 (n=36)				Cycle 4 (n=35)			
	**Mean**	**SD**	**Mean**	**SD**	**%Change**	**p-value**	**Mean**	**SD**	**%Change**	**p-value**	**Mean**	**SD**	**%Change**	**p-value**	**Mean**	**SD**	**%Change**	**p-value**
Anger/irritability	2.921	0.632	1.973	0.726	-32.46%	**<0.0001**	1.892	0.567	-35.23%	**<0.0001**	1.833	0.655	-37.24%	**<0.0001**	1.914	0.742	-34.47%	**<0.0001**
Anxiety/tension	2.842	0.945	2.243	0.830	-21.07%	**0.0111**	2.162	0.898	-23.92%	**0.0058**	2.194	0.920	-22.79%	**0.0123**	1.971	0.785	-30.63%	**<0.0001**
Tearful/increased sensitivity to rejection	2.842	0.823	2.027	0.986	-28.68%	**0.0018**	2.000	0.943	-29.63%	**0.0012**	1.972	0.878	-30.61%	**0.0002**	1.714	0.860	-39.68%	**<0.0001**
Depressed mood/hopelessness	2.658	0.815	2.081	0.894	-21.70%	**0.0138**	1.784	0.750	-32.89%	**<0.0001**	1.889	0.887	-28.93%	**0.0020**	1.771	0.731	-33.35%	**<0.0001**
Decreased interest in work activities	2.447	0.921	1.919	0.722	-21.59%	**0.0042**	1.730	0.871	-29.32%	**0.0004**	1.833	1.028	-25.09%	**0.0361**	1.771	0.808	-27.62%	**0.0103**
Decreased interest in home activities	2.500	0.893	1.892	0.658	-24.32%	**0.0006**	1.676	0.784	-32.97%	**0.0001**	1.778	0.929	-28.89%	**0.0009**	1.800	0.797	-28.00%	**0.0038**
Decreased interest in social activities	2.816	0.955	1.865	0.887	-33.77%	**<0.0001**	1.946	0.911	-30.89%	**<0.0001**	1.778	0.929	-36.86%	**<0.0001**	1.686	0.676	-40.13%	**<0.0001**
Difficulty concentrating	2.316	0.873	1.703	0.777	-26.47%	**0.0004**	1.676	0.884	-27.64%	**0.0039**	1.778	0.959	-23.23%	**0.0410**	1.771	0.808	-23.51%	0.0643
Fatigue/lack of energy	3.289	0.654	2.486	0.837	-24.41%	**<0.0001**	2.297	0.845	-30.16%	**<0.0001**	2.306	0.786	-29.91%	**<0.0001**	2.171	0.954	-33.99%	**<0.0001**
Overeating/food cravings	2.816	0.865	2.162	0.898	-23.21%	**0.0015**	2.243	0.863	-20.33%	**0.0062**	1.889	0.887	-32.92%	**0.0005**	2.000	0.874	-28.97%	**0.0007**
Insomnia	2.184	0.955	1.811	0.845	-17.10%	0.0801	1.703	0.740	-22.04%	**0.0215**	1.611	0.728	-26.24%	**0.0075**	1.514	0.612	-30.67%	**0.0048**
Hypersomnia (needing more sleep)	2.605	1.001	1.757	0.895	-32.57%	**0.0003**	1.811	0.845	-30.49%	**0.0026**	1.889	1.008	-27.50%	**0.0043**	1.829	0.923	-29.81%	**0.0005**
Feeling overwhelmed or out of control	2.526	0.862	1.838	0.764	-27.25%	**0.0005**	1.649	0.716	-34.74%	**<0.0001**	1.639	0.931	-35.13%	**0.0003**	1.600	0.775	-36.67%	**<0.0001**
Physical symptoms: breast tenderness, headaches, joint/muscle pain, bloating, weight gain	3.053	0.695	2.405	0.896	-21.20%	**0.0025**	2.216	0.821	-27.40%	**<0.0001**	2.028	0.736	-33.57%	**<0.0001**	1.914	0.781	-37.29%	**<0.0001**

% Change indicates a change in mean values from baseline. A decrease in score indicates an improvement in the PMS parameter. This table provides the numerical data presented in Figure 1. Green cells highlight statistically significant outcomes. Total number of patients (n) = 34; **SD:** standard deviation.

Table S2: Effect of the FemmeBalance Supplement on Premenstrual Symptom Interference with Daily Activities and Relationships Evaluated by the PSST Questionnaire.

**Have your symptoms, as listed above, interfered with:**	**Baseline (n=38)**		**Cycle 1 (n=37)**				**Cycle 2 (n=37)**				**Cycle 3 (n=36)**				**Cycle 4 (n=35)**			
	**Mean**	**SD**	**Mean**	**SD**	**% Change**	**p-value**	**Mean**	**SD**	**% Change**	**p-value**	**Mean**	**SD**	**% Change**	**p-value**	**Mean**	**SD**	**% Change**	**p-value**
Your work efficiency or productivity	2.553	0.760	1.703	0.702	-33.30%	**<0.0001**	1.568	0.765	-38.59%	**<0.0001**	1.528	0.736	-40.15%	**<0.0001**	1.571	0.698	-38.44%	**<0.0001**
Your relationships with coworkers	2.053	0.985	1.378	0.681	-32.85%	**0.0026**	1.378	0.721	-32.85%	**0.0011**	1.139	0.424	-44.52%	**<0.0001**	1.343	0.591	-34.58%	**<0.0001**
Your relationships with your family	2.526	0.922	1.541	0.767	-39.02%	**<0.0001**	1.378	0.545	-45.44%	**<0.0001**	1.306	0.577	-48.32%	**<0.0001**	1.429	0.655	-43.45%	**<0.0001**
Your social life activities	2.500	0.952	1.757	0.983	-29.73%	**0.0016**	1.703	0.777	-31.89%	**0.0001**	1.500	0.697	-40.00%	**<0.0001**	1.514	0.702	-39.43%	**<0.0001**
Your home responsibilities	2.605	0.823	1.784	0.821	-31.53%	**0.0001**	1.568	0.689	-39.83%	**<0.0001**	1.611	0.803	-38.16%	**<0.0001**	1.600	0.812	-38.59%	**<0.0001**

% Change indicates a change in mean values from baseline. A decrease in score indicates an improvement in the PMS parameter. This table provides the numerical data presented in Figure 2. Green cells highlight statistically significant outcomes. **SD:** standard deviation

Table S3: Effect of the FemmeBalance Supplement on Heaviness and Length of Period Evaluated by Study Specific Questionnaires.

Thinking back to your last period:	Baseline (N=38)		Cycle 1 (N=37)				Cycle 2 (N=37)				Cycle 3 (N=36)				Cycle 4 (N=35)			
	**Mean**	**SD**	**Mean**	**SD**	**%Change**	**p-value**	**Mean**	**SD**	**%Change**	**p-value**	**Mean**	**SD**	**%Change**	**p-value**	**Mean**	**SD**	**%Change**	**p-value**
How heavy was your period?	3.579	0.683	3.081	0.682	-13.91%	**0.0005**	3.135	0.631	-12.40%	**0.0019**	3.111	0.747	-13.07%	**0.007**	2.971	0.891	-16.97%	**0.0004**
How many days did your period last, from the time the bleeding began until it completely stopped?	5.895	1.503	5.649	1.358	-4.17%	0.7156	5.571	1.220	-5.48%	0.8523	5.611	1.460	-4.81%	0.3848	5.629	1.215	-4.52%	0.7945

% Change indicates a change in mean values from baseline. A decrease in score indicates an improvement in the parameter. This table provides the numerical data presented in Figure 3. Green cells highlight statistically significant outcomes; **SD:** standard deviation.

Table S4: Effect of the FemmeBalance Supplement on Premenstrual Symptoms Evaluated by Study-Specific Questionnaires.

In the week before and the week of your last period:	Baseline (n=38)		Cycle 1 (n=37)				Cycle 2 (n=37)				Cycle 3 (n=36)				Cycle 4 (n=35)			
	Mean	SD	Mean	SD	%Change	p-value	Mean	SD	%Change	p-value	Mean	SD	%Change	p-value	Mean	SD	%Change	p-value
How severe were any menstrual pain/cramps you experienced?	3.789	0.843	2.811	1.023	-25.83%	<0.0001	2.649	0.949	-30.11%	<0.0001	2.389	0.903	-36.96%	<0.0001	2.457	0.980	-35.16%	<0.0001
How severe was any pelvic discomfort you experienced?	3.526	0.951	2.622	1.010	-25.66%	0.0004	2.378	0.953	-32.55%	<0.0001	2.194	0.980	-37.77%	<0.0001	2.114	1.078	-40.04%	<0.0001
How severe were any mood swings you experienced?	3.658	0.847	2.757	0.955	-24.64%	<0.0001	2.351	0.949	-35.72%	<0.0001	2.417	0.937	-33.93%	<0.0001	2.114	1.078	-42.20%	<0.0001
How severe was any hormonal acne you experienced?	3.105	1.085	2.108	0.843	-32.11%	<0.0001	2.216	1.158	-28.63%	<0.0001	2.278	0.974	-26.65%	0.0001	2.286	1.152	-26.39%	0.0073
How severe was any hormonal hair loss you experienced?	2.316	1.317	1.811	1.175	-21.81%	0.0659	1.514	0.961	-34.64%	0.0019	1.722	1.003	-25.63%	0.0484	1.657	1.110	-28.44%	0.057
How severe was any fluid retention or bloating you experienced?	3.553	0.686	2.730	0.990	-23.16%	<0.0001	2.216	1.004	-37.62%	<0.0001	2.194	0.920	-38.23%	<0.0001	2.114	1.132	-40.49%	<0.0001
How severe was any breast pain or tenderness you experienced?	3.105	1.008	2.000	1.000	-35.59%	<0.0001	1.865	1.134	-39.95%	<0.0001	1.806	0.856	-41.85%	<0.0001	1.686	0.796	-45.71%	<0.0001
How severe was any irritability you experienced?	3.816	0.730	2.730	0.962	-28.46%	<0.0001	2.054	0.941	-46.17%	<0.0001	2.250	1.025	-41.03%	<0.0001	2.257	0.980	-40.85%	<0.0001
How severe were any episodes of crying or sadness you experienced?	3.289	1.037	2.378	1.063	-27.70%	0.001	2.135	0.976	-35.09%	<0.0001	2.194	1.142	-33.29%	0.0002	2.057	1.083	-37.46%	0.0001
How severe was any emotional distress you experienced?	3.421	0.976	2.568	1.068	-24.95%	0.0014	2.189	1.050	-36.01%	<0.0001	2.222	1.198	-35.04%	0.0002	2.086	1.095	-39.03%	<0.0001

% Change indicates a change in mean values from baseline. A decrease in score indicates an improvement in the PMS parameter. This table provides the numerical data presented in Figure 4. Green cells highlight statistically significant outcomes; **SD:** standard deviation.

Table S5: Effect of the FemmeBalance Supplement on Premenstrual Symptoms Evaluated by Study-Specific Questionnaires.

In the week before and the week of your last period:	Baseline (n=38)		Cycle 1 (n=37)				Cycle 2 (n=37)				Cycle 3 (n=36)				Cycle 4 (n=35)			
	**Mean**	**SD**	**Mean**	**SD**	**%Change**	**p-value**	**Mean**	**SD**	**%Change**	**p-value**	**Mean**	**SD**	**%Change**	**p-value**	**Mean**	**SD**	**%Change**	**p-value**
How would you rate your well-being?	3.816	0.730	3.784	0.630	-0.84%	0.9981	3.811	0.518	-0.13%	>0.9999	3.833	0.561	0.46%	0.9987	3.857	0.648	1.08%	0.9890
How would you rate your overall menstrual health?	3.316	0.933	3.784	0.787	14.11%	0.0170	3.595	0.762	8.41%	0.1999	3.833	0.609	15.61%	0.0050	3.857	0.648	16.33%	0.0353
How would you rate your hormonal balance?	3.237	0.883	3.459	0.836	6.88%	0.5860	3.568	0.765	10.22%	0.2587	3.639	0.593	12.42%	0.0917	3.686	0.796	13.87%	0.0956
How would you rate your hair health?	3.421	0.948	3.514	1.044	2.70%	0.9393	3.595	0.985	5.07%	0.7746	3.611	0.934	5.56%	0.5465	3.686	0.963	7.74%	0.5453
How would you rate your skin health?	3.316	0.842	3.622	0.861	9.22%	0.1537	3.676	0.884	10.85%	0.0687	3.750	0.770	13.10%	0.0212	3.657	0.802	10.29%	0.0681

% Change indicates a change in mean values from baseline. An increase in score indicates an improvement in the PMS parameter. This table provides the numerical data presented in Figure 5. Green cells highlight statistically significant outcomes; **SD:** standard deviation.
